# A systematic review of transcriptomic studies of the human endometrium reveals inconsistently reported differentially expressed genes

**DOI:** 10.1530/RAF-22-0115

**Published:** 2023-07-07

**Authors:** Evangeline R Walker, Mollie McGrane, John D Aplin, Daniel R Brison, Peter T Ruane

**Affiliations:** 1 Department of Reproductive Medicine, Old Saint Mary’s Hospital, Manchester University NHS Foundation Trust, Manchester Academic Health Science Centre, Manchester, UK; 2 Maternal and Fetal Health Research Centre, Division of Developmental Biology and Medicine, School of Medical Sciences, Faculty of Biology, Medicine and Health, University of Manchester, Manchester, UK; 3 Maternal and Fetal Health Research Centre, Saint Mary’s Hospital, Manchester University NHS Foundation Trust, Manchester Academic Health Sciences Centre, University of Manchester, Manchester, UK

**Keywords:** assisted reproduction, uterus, molecular biology of reproduction, women’s health

## Abstract

Genome-wide analysis of gene expression has been widely applied to study the endometrium, although to our knowledge no systematic reviews have been performed. Here, we identified 74 studies that described transcriptomes from whole (unprocessed) endometrium samples and found that these fitted into three broad investigative categories: endometrium across the menstrual cycle, endometrium in pathology and endometrium during hormone treatment. Notably, key participant information such as menstrual cycle length and body mass index was often not reported. Fertility status was frequently not defined and fertility-related pathologies, such as recurrent implantation failure (RIF) and recurrent pregnancy loss, were variably defined, while hormone treatments differed between almost every study. A range of 1307–3637 reported differentially expressed genes (DEGs) were compared in four to seven studies in five sub-categories: (i) secretory vs proliferative stage endometrium, (ii) mid-secretory vs early secretory stage endometrium, (iii) mid-secretory endometrium from ovarian stimulation-treated participants vs controls, (iv) mid-secretory endometrium from RIF patients vs controls, and (v) mid-secretory eutopic endometrium from endometriosis patients vs controls. Only the first two sub-categories yielded consistently reported DEG between ≥3 studies, albeit in small numbers (<40), and these were enriched in developmental process and immune response annotations. This systematic review, though not PROSPERO registered, reveals that limited demographic detail, variable fertility definitions and differing hormone treatments in endometrial transcriptomic studies hinder their comparison, and that the large majority of reported DEG do not advance the identification of underlying biological mechanisms. Future studies should apply network biology approaches and experimental validation to establish causal gene expression signatures.

**Lay summary:**

The endometrium lines the inner wall of the uterus and is the site where the fertilised egg implants to establish pregnancy. Disorders of the endometrium cause infertility and chronic pain. Techniques to measure genetic activity, termed transcriptomics, have been applied to better understand the endometrium in health and disease. We collated all studies, totalling 74, that describe transcriptomics of endometrial samples from non-pregnant women and compared study designs and genetic activity measurements. The studies generally looked at small numbers of samples, with most focussing on fertility rather than endometrial disorders. Study designs were variable, comparing women under different definitions of fertility and disease, and under different treatments. Additionally, key participant factors such as BMI were mostly not reported. These and other limitations produced genetic activity measurements that were inconsistent, especially in cases of infertility and endometrial disorders. Addressing these limitations could improve how transcriptomic approaches are used to advance endometrial health.

## Introduction

The endometrium has evolved to become a highly dynamic tissue in response to hormones produced throughout the ovarian cycle, enabling the deep placentation required for human fetal development (Wagner *et al.* 2014). During the proliferative phase, the endometrium undergoes oestrogen-mediated expansion over ~10 days, then differentiates through the progesterone-driven secretory phase for ~14 days and, in the absence of a pregnancy, breaks down over several days after progesterone withdrawal in the menses phase (Aplin *et al.* 2008). Biologists, gynaecologists and obstetricians have long sought to understand the menstrual cycle to improve women’s and children’s health (Critchley *et al.* 2020), and the interest in characterising the endometrium has been driven by assisted reproductive technologies (ART) that rely on the functionality of the mid-secretory phase for embryo implantation and ongoing pregnancy (Craciunas *et al.* 2019).

The basalis layer of the endometrium contains glandular and stromal compartments and develops through the proliferative phase to form the upper functionalis layer (Ferenczy & Bergeron, 1991). Tall glands form in the functionalis (Tempest *et al.* 2022), and stromal fibroblasts proliferate and populate the extracellular matrix to structure the tissue (Salamonsen 2003). Arteries that grow in the proliferative phase as the tissue thickens take on a tortuous spiral shape in the secretory phase (Farrer-Brown *et al.* 1970), and both myeloid and lymphoid immune cells, especially natural killer cells, are recruited from the bloodstream (Wang *et al.* 2021). Thus, maturation in response to progesterone in the mid-secretory endometrium provides the multi-layered substrate to support embryo attachment and invasion at implantation and to shape ingression of extra-embryonic tissues during placentation (Gellersen & Brosens 2014). Endometrial pathologies are a significant burden to women across the world, with chronic pain and infertility among the symptoms of non-malignant conditions including endometriosis, adenomyosis and fibroids (Driva *et al.* 2022). Recurrent implantation failure after ART treatment has also been postulated to be at least partly caused by endometrial dysfunction (Santamaria & Simon 2021), as has recurrent miscarriage in naturally conceived and ART pregnancies (Brosens *et al.* 2022). Moreover, there is a growing body of evidence that endometrial dysfunction during placentation underlies obstetric disorders such as preeclampsia, fetal growth restriction and stillbirth (Conrad 2020).

To understand the cellular processes governing endometrial biology and how dysfunction may lead to pathology, one avenue of research over the last two decades has attempted to define the endometrium at the genomic level using nucleotide sequencing technologies. This approach aims to provide a view of the totality of expressed genes in a sample (the transcriptome) following the hypothesis that detectable gene expression differences will explain tissue function and disorder (Diaz-Gimeno *et al.* 2014). Initially, nucleotide hybridisation technologies, or microarrays, supported transcriptomic studies, but in the last decade, direct RNA sequencing has enabled much higher depth analysis. More recently, single-cell-resolution transcriptomics has enabled distinct cell populations within the endometrium to be identified based on their transcriptome, and their development and interactions interrogated in exquisite detail (Wang *et al.* 2020, Garcia-Alonso *et al.* 2021, Stevens *et al.* 2023). A much more fine-grained understanding of endometrial biology has been characterised in this way; however, this increased resolution comes at the cost of limited sample sizes, and so single-cell transcriptomics is yet to be used efficiently to interrogate endometrial dysfunction in patients.

Molecular signatures of functionality and pathology have been sought in ‘bulk’ (not single-cell resolution) endometrial transcriptomic studies based on differential expression of genes between superficial biopsy samples. Attempts to identify a mid-secretory endometrial state receptive to embryo implantation have fed through to clinical application during ART (Gomez *et al.* 2015). The endometrial receptivity array (ERA) entails analysis of 279 specific genes derived from bulk transcriptomic studies and has been used to test (in a previous cycle) for the optimal time during the mid-secretory phase for embryo transfer in an ART cycle (Diaz-Gimeno *et al.* 2013). The efficacy of this approach, however, has been questioned (Tan *et al.* 2018, Cohen *et al.* 2020, Ben Rafael 2021) and recently exposed as actually reducing success rates (Richter & Richter 2023), while newer approaches using whole transcriptome assessments supported by machine learning may also be ineffective (Aplin & Stevens 2022).

As costs for transcriptome sequencing continue to fall, there are likely to be wider applications to ART and novel incursions for clinically assessing gynaecological conditions. Reviewing previous studies may provide clues as to which pathways might be biomarkers or even druggable, and previous meta-analyses of the endometrial transcriptome literature have robustly identified key molecular pathways underlying the secretory phase (Altmae *et al.* 2017) and endometriotic disease (Poli-Neto *et al.* 2020, Vargas *et al.* 2022). However, a systematic review of the totality of bulk endometrial transcriptomic studies is not available and could provide evidence on the comparability of such studies, the utility of bulk transcriptomics and analysis based on differential gene expression between samples in identifying functional differences, as well as insight into the biology underpinning endometrial function and pathology.

## Methods

### Literature search

Literature searches were performed to identify studies that fitted the inclusion criteria: analysis of the transcriptome of whole biopsy samples of human endometrium. The following search terms were used in PubMed: (‘endomet*’ OR ‘endometrium’ OR ‘endometrial’ OR ‘uterine’ OR ‘uterus’) AND (‘transcrip*’ OR ‘transcriptome’ OR ‘gene expression’ OR ‘gene profile’ OR ‘RNA’) AND (‘sequenc*’ OR ‘sequencing’ OR ‘RNAseq’ OR ‘microarray’ OR ‘array’ OR ‘cDNA’ OR ‘Affymetrix’ OR ‘Agilent’). A filter was applied to include only English language studies with human participants (confirmed by assessment of titles and abstracts). Additional studies were identified by examining reference lists of review articles and co-authors’ data and knowledge bases. Exclusion criteria included the use of non-human tissue, use of gene-directed PCR, decidual or placental tissue sampling, meta-analyses, use of the ERA/ERmap, cultured endometrial cells, analysis using enriched/sorted cells and post-menopausal samples. This systematic review was not PROSPERO registered.

### Extraction of metadata from studies

Full-text articles of studies which met the inclusion criteria were assessed, and metadata were compiled for each study. Criteria were determined based on the assessment of review articles describing common methodologies of these studies (Altmäe *et al.* 2014).

### Differential gene expression comparisons

DEGs were compared between studies where the DEG lists were accessible in the published manuscript or associated supplementary data. Studies were included where a sub-analysis provided a relevant assessment and DEG information. Curation of the DEG lists to ensure consistent gene nomenclature was performed using online tools G:Prolifer (Raudvere *et al.* 2019) and DAVID v6.8 (Huang *et al.* 2009).

Commonly reported DEGs (cDEGs) were collated with associated numerical fold-change where available (DEG reported with no numerical fold-change were utilised in determining common reportage). Where genes had been reported as only up- or down-regulated with no numerical fold-change, these contributed to the classification of cDEG as consistently or inconsistently differentially expressed between studies. The average fold-changes of cDEG between studies were calculated where available.

### Gene ontology analysis

Overrepresentation of Biological Processes was assessed for cDEG lists using WebGestalt (Zhang *et al.* 2005).

## Results

### Systematic review of the human whole endometrium transcriptome literature

**Figure 1:**
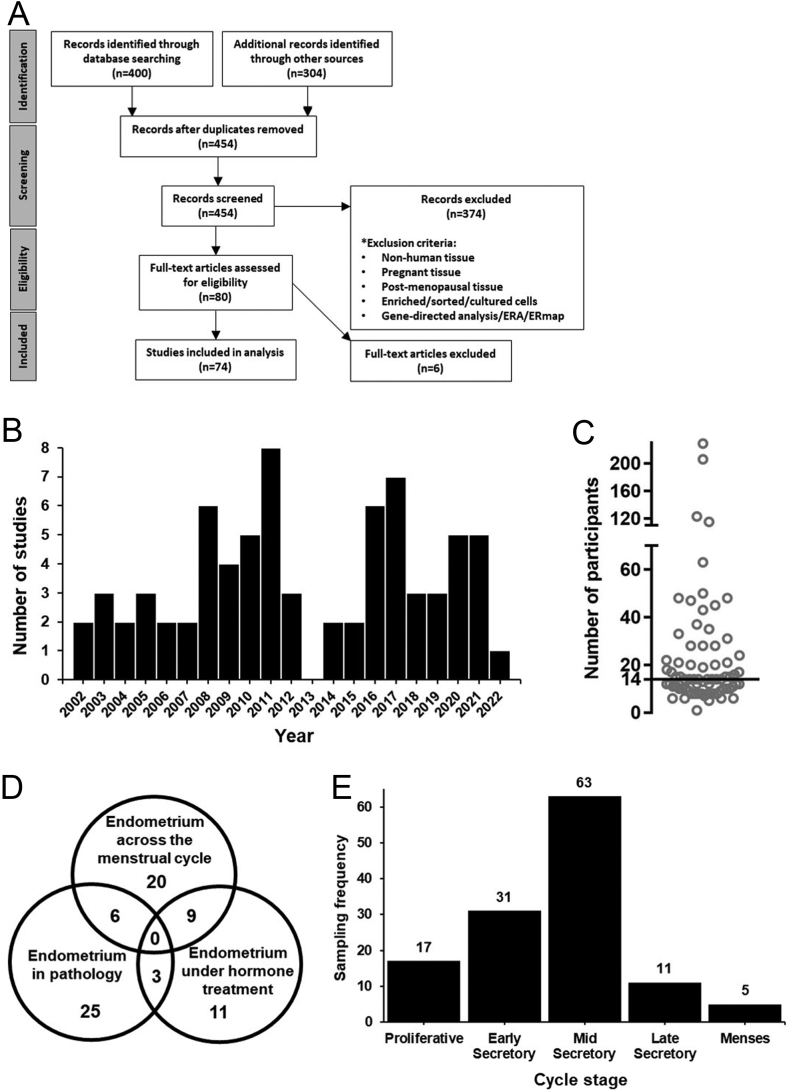
Systematic review process and the characteristics of studies meeting the inclusion criteria. (A) Flow diagram of the systematic review process for this study, culminating in 74 studies that met the inclusion criteria. (B) Plot of the number of included studies published in each year of the review period. (C) Plot of the number of participants in each of the included studies. The median number of participants is indicated by a black line. (D) Venn diagram indicating how the included studies fit within three investigative categories. (E) Plot of the frequency of menstrual cycle stages sampled in 72/74 included studies (cycle stage not reported in two studies).

A total of 454 studies published between January 2002 and August 2022 that mentioned endometrial transcriptomic approaches were identified and screened, leading to 70 studies that were found to have analysed the transcriptome of whole human eutopic endometrial samples (Fig. 1A and B). Supplementary Table 1 (see section on supplementary materials given at the end of this article) lists the 74 studies with associated metadata. There was a range in size for these studies, with the largest analysing 229 participant transcriptomes and the smallest looking at one participant transcriptome; the median number of participants was 14 (Fig. 1C). The large majority of studies (57/74) analysed transcriptome by microarray, with only 17/74 using RNAseq. Transcriptome data were available with the manuscript or deposited in an online database in just 30/74 studies.

The primary aims of these studies could be broken down into four investigative categories: to define the endometrial transcriptome at different stages of the menstrual cycle (*n* = 20), in pathology (*n* = 25), under hormone treatment (*n* = 11) and combinations of these categories (menstrual cycle stage and pathology, *n* = 6; menstrual cycle stage and hormone treatment, n = 9; pathology and hormone treatment, *n* = 3) (Fig. 1D, Supplementary Table 1). The full range of the menstrual cycle was sampled across all of the studies, with the majority focusing on the mid-secretory phase. Notably, menses phase endometrium was only sampled in five groups among all of these studies (Fig. 1E). Most studies (51/74) used biochemical definitions of cycle stage (LH/hCG), with the remainder (19/74) using histology (e.g. Noyes criteria), ultrasound scanning (3/74) or self-reported last menstrual period (1/74). Where reported, participant menstrual cycle length was between 25 and 35 days in 22 studies, with a further 16 studies reporting cycle length to be ‘normal’ or ‘regular’, while participants with cycle length of >35 days were included in four studies. Participant menstrual cycle length was not reported in 30/74 studies.

### Participant demographics

Almost half of the included studies took place in Europe (35/74); others took place in North America (17/74), Asia (13/74), Australasia (5/74) or Central/South America (4/74). No studies were conducted in Africa. The age of participants was reported in 67/74 studies and was 18–48 years. Ethnicity, body mass index (BMI) and parity of participants were not reported in the large majority of studies (58/74, 47/74 and 64/74, respectively). Ethnicity of participants was Caucasian only in four studies, four studies included participants of European ancestry only, another study included Hispanic participants only and the remaining seven studies that reported ethnicity cited a mixed participant population, including mixed ethnicity, Asian, Black, Asian-Indian, Latina, African-American, White, Hispanic, Caucasian and unknown. Of those studies that reported BMI, 15/27 reported all participants to be within the healthy range of 18.5–24.5 kg/m^2^, while 12/27 included participants ≥25 kg/m^2^. Endometrial parameters described in some studies were thickness (given in 7/74 studies), location of sampling (20/74) and method of sampling (52/74).

### Pathology definitions

**Figure 2:**
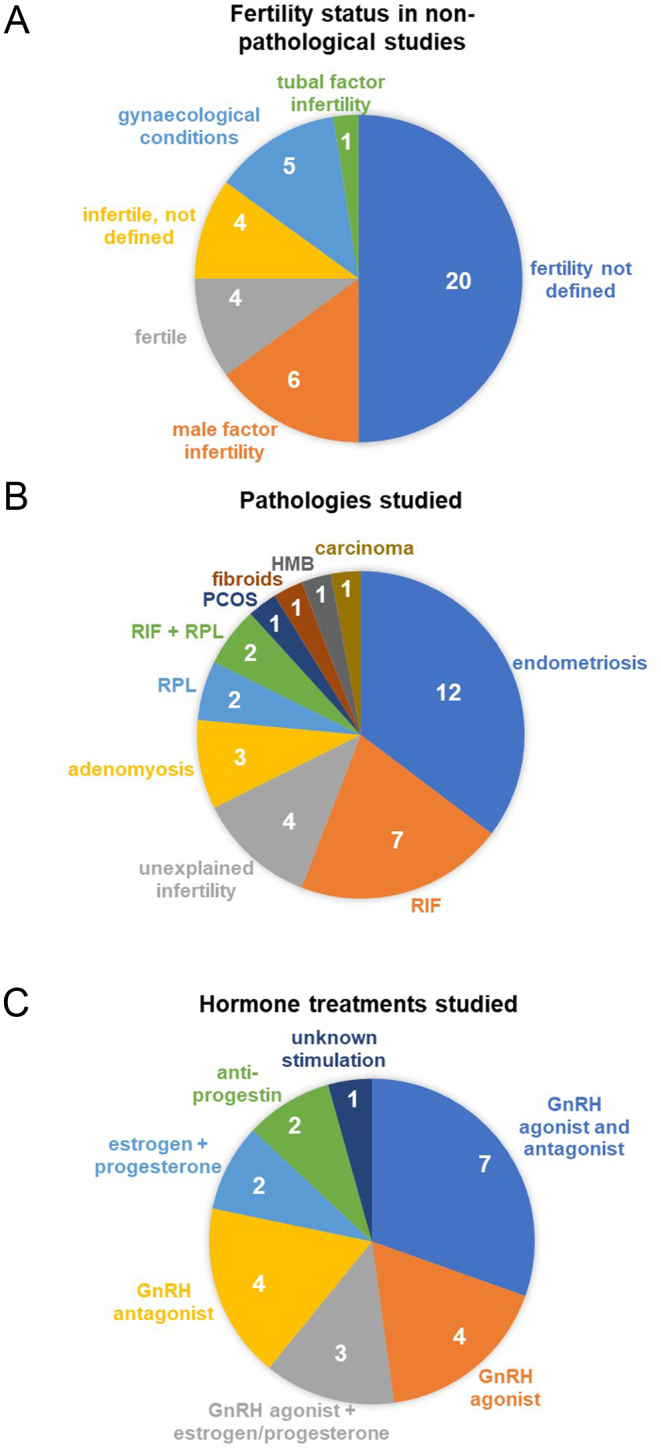
Definitions and characteristics of investigative categories. (A) Pie chart indicating the fertility definitions reported in non-pathologically included studies. (B) Pie chart showing the pathologies in the included studies. (C) Pie chart representing the hormone treatments used in the included studies.

The majority of the studies identified in this systematic review (40/74) did not investigate pathology as a primary outcome. Of these, half (20/40) did not define any pathology in the participants although many were seeking fertility treatment, 4/40 describe participants as fertile (specifically defined with parity in 2/4 studies), 6/40 describe participants as seeking fertility treatment due to male factor infertility, 1/40 describe participants as having tubal factor infertility, 4/40 describe participants as infertile with no other information and the remaining 5/40 describe participants as having a range of gynaecological conditions encompassing endometriosis, uterine fibroids, uterine prolapse, endometrial polyps, endometrial carcinoma, ovarian cyst and pelvic pain (Fig. 2A).

In those studies that evaluated the effect of a pathology on the endometrial transcriptome as a primary outcome, approaching half (15/34) looked at pathologies of fertility, unexplained fertility (4/34), rapid implantation failure (RIF; 7/3 4), recurrent pregnancy loss (RPL; 2/34) and RIF and RPL (2/34). Over a third of the pathology studies investigated endometriosis (12/34), with the remainder investigating adenomyosis (3/34), polycystic ovary syndrome (PCOS) (1/34), uterine fibroids (1/34), heavy menstrual bleeding (HMB) (1/34) and endometrial carcinoma (1/34) (Fig. 2B). A range of definitions for these pathologies were applied; RIF was defined by various methods as summarised in the following equation:


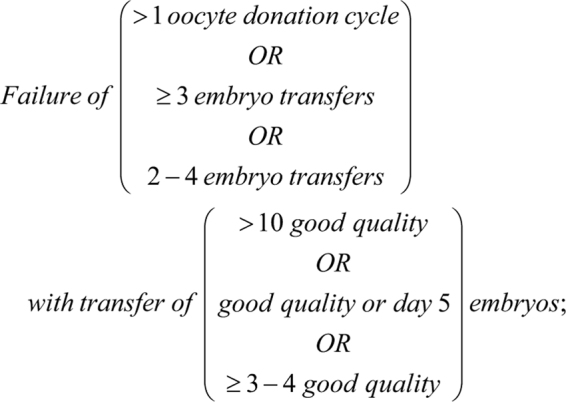



Endometriosis was as defined by the American Fertility Society/Society for Reproductive Medicine classification scheme (The American Fertility Society 1985, American Society for Reproductive Medicine 1997) (6/12) or not specifically defined (6/12); RPL was defined variously as ≥2 miscarriages under 10 weeks, ≥2 or ≥3 consecutive miscarriages between 6 and 12 weeks or ≥3 consecutive miscarriages under 24 weeks; unexplained fertility was defined by ICMART (Zegers-Hochschild *et al.* 2017) or not specifically defined; PCOS was defined by Rotterdam criteria (Rotterdam ESHRE/ASRM-Sponsored PCOS Consensus Workshop Group 2004); adenomyosis and fibroids were determined by histological analysis not relating to any specific definitions; HMB was established by patient ‘complaint’; and endometrial cancer was defined by FIGO staging (Creasman 2009).

### Hormone treatments

Twenty-three studies investigated the effects of hormone treatment on endometrial transcriptomes, typically as part of ovarian or endometrial stimulation in ART cycles. The large majority of these looked at ovarian stimulation using gonadotrophin-releasing hormone (GnRH) agonist treatment (7/23), GnRH antagonist treatment (4/23),or both (7/23), with one study not detailing the ovarian stimulation treatment given. Endometrial stimulation with oestrogen and/or progesterone was analysed in natural ovarian cycles in two studies and in conjunction with GnRH agonist ovarian stimulation in three studies. The remaining studies of the endometrial transcriptome under hormone treatment investigated the effects of progestin contraceptives (Fig. 2C). There was a great deal of variation in hormone regime between studies, but all sampled the endometrium in the secretory phase.

### Five common comparisons reporting differentially expressed genes within the whole endometrium transcriptome literature

Five common comparisons that were amenable to analysis of reported (DEGs were identified from the systematic review of the literature: (i) secretory vs proliferative stage in ‘normal’ participants (five studies), (ii) mid-secretory vs early secretory stage in ‘normal’ participants (seven studies), (iii) mid-secretory from ovarian stimulation-treated participants vs controls (six studies), (iv) mid-secretory from RIF patients vs controls (seven studies), and (v) mid-secretory eutopic endometrium from endometriosis patients vs controls (four studies). These comparisons represent 27 distinct studies, with a range of 53–233 participants and 1307–3637 DEG (Table 1).

**Table 1:** Studies of common comparisons which reported differentially expressed genes and associated cumulative metadata.

Comparison	Studies, *n*	Partici-pants, *n*	Total Reported DEG	Studies
Secretory vs proliferative	5	80	3637	Kao *et al.* (2002), Borthwick *et al.* (2003), Talbi *et al.* (2006), Otsuka *et al.* (2007), Sigurgeirsson *et al.* (2017)
Mid-secretory vs early secretory	7	112	2795	Carson *et al.* (2002), Riesewijk *et al.* (2003), Mirkin *et al.* (2005), Talbi *et al.* (2006), Haouzi *et al.* (2009), Tseng *et al.* (2010), Hu *et al.* (2014)
Mid-secretory endometrium from hormone-treated participants vs controls	6	112	1937	Mirkin *et al.* (2004), Horcajadas *et al.* (2005, 2008), Liu *et al.* (2008), Macklon *et al.* (2008), Li *et al.* (2020)
Mid-secretory endometrium from RIF patients vs controls	7	233	1651	Tapia *et al.* (2008), Koler *et al.* (2009), Ledee *et al.* (2011), Choi *et al.* (2016), Koot *et al.* (2016), Bastu *et al.* (2019), Chen *et al.* (2019)
Mid-secretory eutopic endometrium from endometriosis patients vs controls	4	53	1307	Kao *et al.* (2003), Burney *et al.* (2007), Sherwin *et al.* (2008), Cui *et al.* (2018)

### Inconsistent differential expression and limited similarity between commonly reported DEG

**Figure 3:**
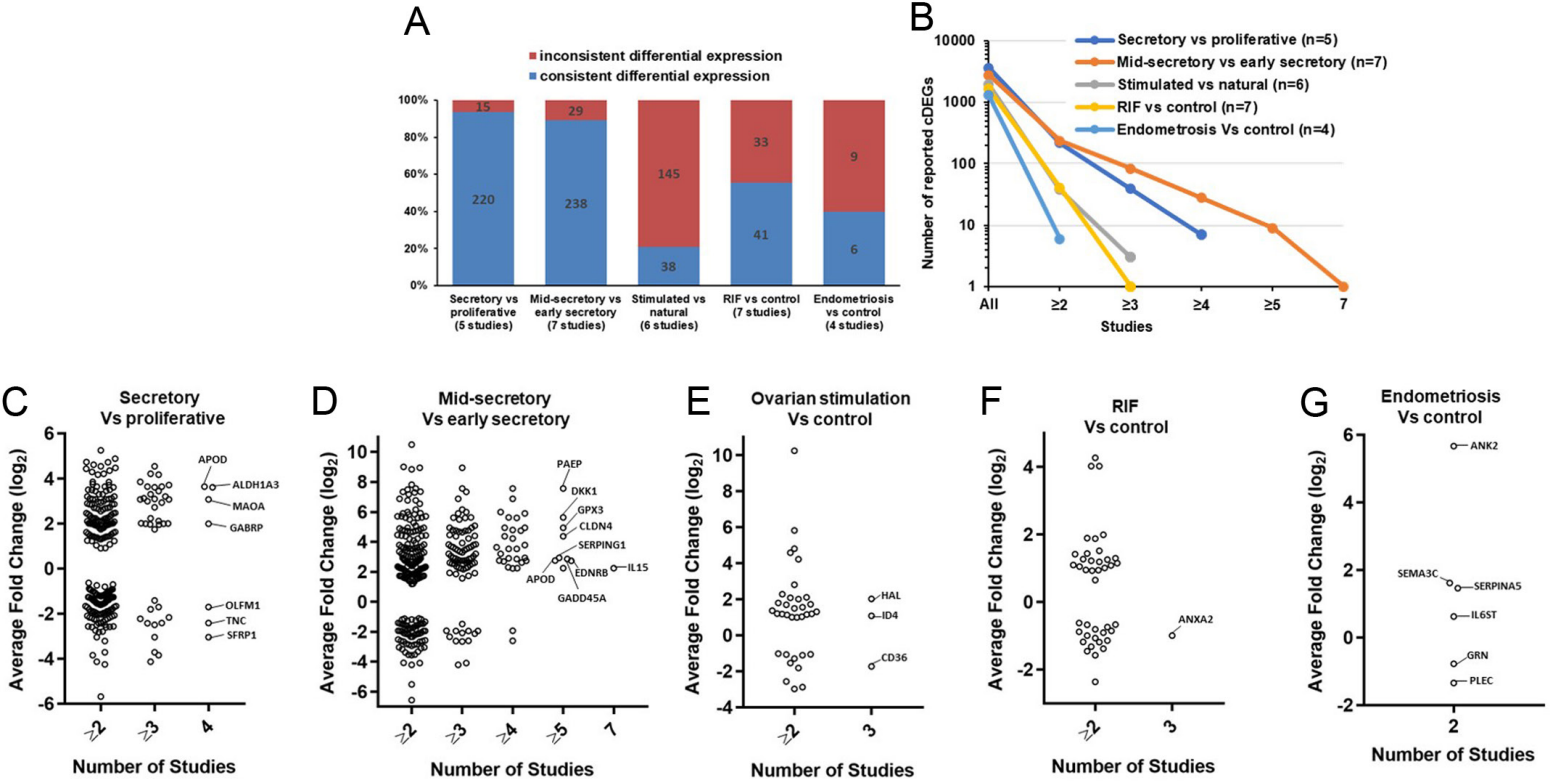
Patterns of differentially expressed gene reporting in common comparisons among studies. (A) The proportion of commonly reported differentially expressed genes (cDEG) across ≥2 studies for each comparison that show consistent (blue) and inconsistent (red) directionality of change in expression. Number of cDEG is indicated in each bar. (B) The number of consistently changed cDEG for each comparison plotted against number of studies in which they are commonly reported. (C–G) Dot plots of consistently changed cDEG fold-change averaged between studies for each comparison. The most commonly reported cDEG gene names are indicated on the plots.

cDEG across ≥2 studies for each comparison were identified and the fold-change of expression in each study was compared. A notable minority of cDEG were found to have inconsistent directionality of change (i.e., reported as up- or down-regulated) between two or more studies in secretory vs proliferative endometrium and mid-secretory vs early secretory endometrium comparisons (6.8% and 10.9%, respectively) (Fig. 3A). Strikingly high rates of inconsistent directionality of change were found for cDEG in studies comparing mid-secretory endometrium from ovarian stimulation-treated participants vs controls (79.2%), mid-secretory endometrium from RIF patients vs controls (45%) and mid-secretory endometrium from endometriosis patients vs controls (60%) (Fig. 3A). cDEG exhibiting inconsistent directionality of change were not included in subsequent analyses.

The number of cDEG decreased dramatically as the number of studies increased for each comparison (Fig. 3B). Only 6 cDEGs were found between 2/4 studies comparing mid-secretory endometrium from endometriosis patients vs controls, from a total of 1307 DEG. Less than 5 cDEG were found between 3 studies comparing mid-secretory endometrium from ovarian stimulation-treated participants vs controls and those comparing mid-secretory endometrium from RIF patients vs controls (total of 1937 DEG from 6 studies and 1651 DEG from 7 studies, respectively) (Fig. 3B). Studies comparing mid-secretory vs early secretory endometrium plotted a less sharply declining trajectory of cDEG; 28 between 4 or more studies, 9 between 5 or more studies and 1, *IL15*, between all 7 studies from a total of 2795 DEG (Fig. 3B). The cDEG from studies comparing secretory vs proliferative endometrium followed an intermediate trajectory, with 39 and 7 cDEG between 3 and 4 studies, respectively, from a total of 3637 DEG across 5 studies (Fig. 3B). The spread of cDEG average differential expression is shown in Fig. 3C, D, E, F, and G. Notably, there was a strong bias towards up-regulation of genes in studies comparing mid-secretory vs early secretory stage endometrium, with 85%, 93% and 100% cDEG up-regulated in ≥3, ≥4 and ≥5 studies, respectively (Fig. 3D).

### Functional annotations present in cDEG between menstrual cycle stages but not hormone treated or RIF endometrium

Gene ontology analysis was applied to identify functional annotations in 39 cDEG common to ≥3 studies in the comparisons of secretory vs proliferative endometrium (Supplementary Table 2), 28 cDEG common to ≥4 studies comparing mid-secretory vs early secretory endometrium (Supplementary Table 3), 38 cDEG common to ≥2 studies comparing mid-secretory endometrium from ovarian stimulation-treated participants vs controls (Supplementary Table 4) and 41 cDEG common to ≥2 studies comparing mid-secretory endometrium from RIF patients vs controls (Supplementary Table 5).

**Figure 4:**
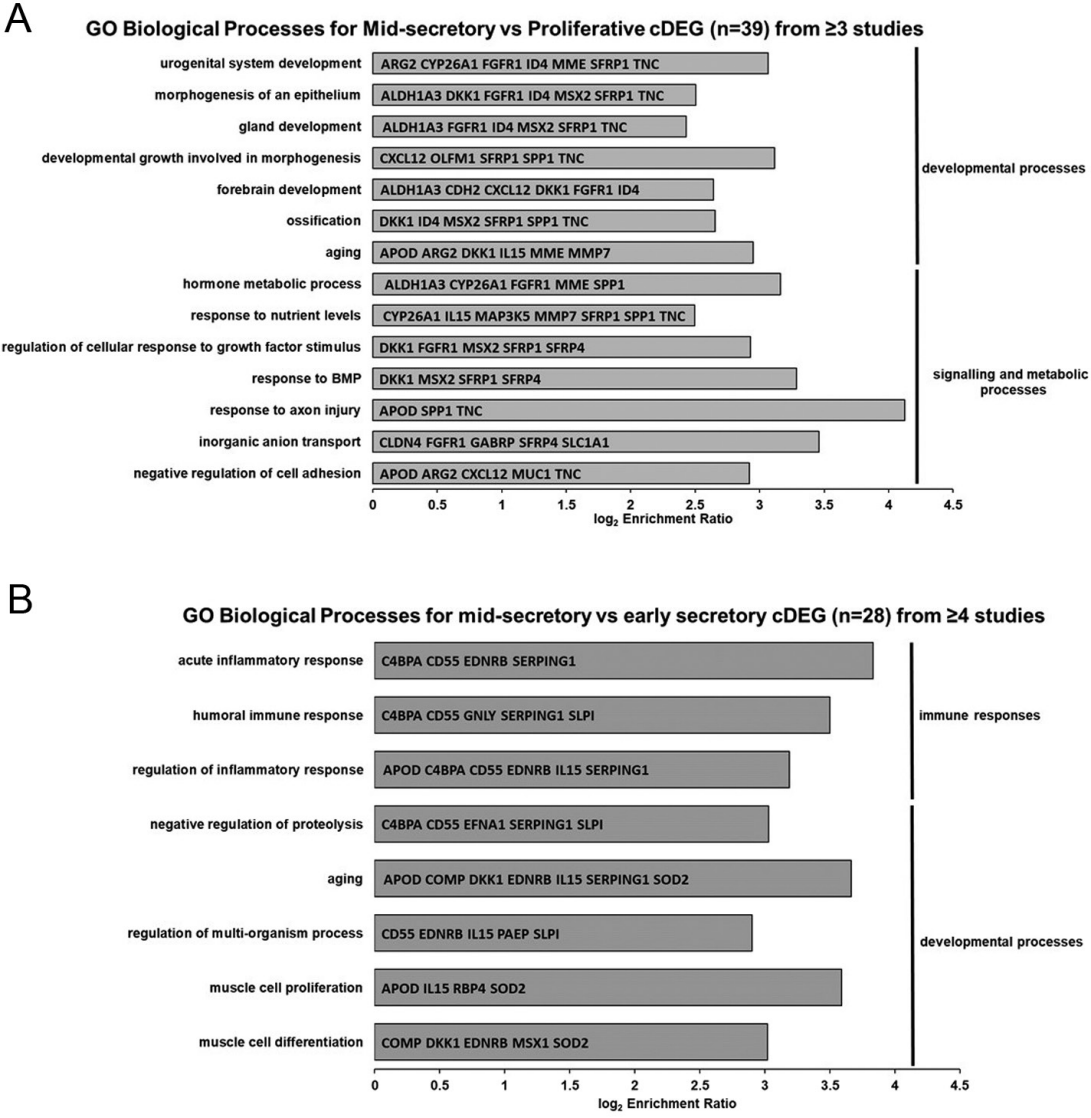
Gene ontology annotations of commonly reported differentially expressed genes. (A) All significantly enriched biological processes within the 39 commonly reported differentially expressed genes (cDEG) from ≥3 studies of secretory vs proliferative endometrium (FDR <0.05). Biological processes are split into broad categories: development process and signalling and metabolic processes. cDEG gene names enriched for these processes are indicated in the bars. (B) All significantly enriched biological processes within the 28 cDEG from ≥4 studies of mid-secretory vs early secretory endometrium (FDR <0.05). Biological processes are split into broad categories: immune response and development process. cDEG gene names enriched for these processes are indicated in the bars.

Remarkably, no functional annotations were found to be enriched in the cases of the ovarian stimulation vs control and RIF vs control analyses. However, 14 and 8 significantly enriched biological processes were identified in secretory vs proliferative endometrium and mid-secretory vs early secretory gene networks, respectively (false discovery rate (FDR) <0.05) (Fig. 4). Enrichment of developmental processes specific to the reproductive tract and to epithelium and gland development, as well as signalling and metabolic processes including hormone metabolism and bone morphogenic protein (BMP) signalling, were present in cDEG from comparison of secretory vs proliferative endometrium (Fig. 4A). cDEGs from the comparison of mid-secretory vs early secretory endometrium were also enriched for developmental processes, although distinct from those seen for secretory vs proliferative gene networks except for a similar aging annotation. However, this comparison exhibited significant enrichment for immune responses, especially inflammatory response (Fig. 4B).

## Discussion

Confirming that transcriptomic analysis has become a common tool for endometrial research, this systematic review found more than 400 studies published over the last two decades that described this approach. We identified 74 studies that analysed the transcriptome of whole human endometrial samples, and these studies fitted into three broad categories of investigation: endometrium across the menstrual cycle, endometrium in pathology and endometrium under hormone treatment. Comparing DEG between studies allowed for a wide analysis of gene level data in this literature. We found a striking pattern of inconsistency between the 27 studies within these categories, most pronounced among those examining endometrium during ovarian stimulation or in the pathologies RIF and endometriosis. Only one gene from a range of 1307–3637 DEG was consistently reported across all comparable studies to be differentially expressed; *IL15* was up-regulated on average 4.77-fold in mid-secretory compared to early secretory endometrium across seven studies. Gene networks involved in developmental and inflammatory processes were apparent among cDEG from ≥3 studies comparing endometrium across the menstrual cycle; however, no functionally linked gene networks were uncovered among any cDEG from any other category. This analysis calls into question the value of transcriptomic studies of the endometrium reporting DEG as the primary outcome.

As direct investigations of the endometrium, almost all included studies here clearly defined the menstrual cycle stage of collection; however, two-fifths of the studies did not report cycle length. Participant age was consistently reported, while other factors known to be linked to menstrual health, BMI and ethnicity (Bull *et al.* 2019) were not described in the large majority of studies. Ethnicity was homogenous in about half the studies that reported it, and almost three-quarters of the studies were performed in Europe and North America; none were performed in Africa. Together, this suggests that our understanding of the endometrial transcriptome can only really be defined with regard to menstrual cycle stage, outside of the context of the complete menstrual cycle in European and North American populations. Endometrial studies are therefore no exception to the heavy bias towards such populations in most genomic fields (Sirugo *et al.* 2019). Superficial biopsies of the functionalis also predominated, and so this literature provides no characterisation of the basalis layer, which could be addressed by samples from hysterectomies. Moreover, investigations into fertility, in terms of normal function, pathology and during ART cycles, are predominant, while endometriosis was the most prominent pathology studied. Fertility, however, was not consistently defined and this limits the reliability of conclusions drawn from comparisons between studies; half of the investigations into basic endometrial biology lacked any description of participant fertility status despite many having received fertility treatment, and when fertility-related pathologies were directly assessed, definitions were not standardised in either the patient or control groups. Inclusion of detailed participant histories may not have complemented early discovery science studies of endometrial transcriptomics but inclusion in later comparative studies, especially those of pathology, improves the evidential quality of the findings.

Endometrial variability has been characterised between samples (Suhorutshenko *et al.* 2018), between menstrual cycles (Evans *et al.* 2018) and between individuals (Bull *et al.* 2019). Studies comparing large endometrial transcriptome datasets from an average of 14 participants are therefore likely to suffer low signal to noise ratio in detecting mechanistic DEG, and comparing DEG between studies will inevitably compound these limitations. Meta-analysis approaches allow for some batch correction between studies to produce a less error-prone comparison of transcriptomic data (Leek *et al.* 2010), but these are constrained by available raw data and comparability between sequencing technologies. A wide but potentially more error-prone comparison of the literature was afforded by comparing reported DEG; however, this can be further confounded by variable study design. This was a particular problem among investigations of the mid-secretory endometrium under ovarian stimulation, in which very few cDEG were identified. Only two of these six studies used the same ovarian stimulation regimen and thus reported DEG may reflect the specific treatments amongst other demographic and technical variables. Moreover, the very high proportion of DEG showing inconsistent directionality of change in this category suggests endometrial transcriptomic differences may be quite limited and therefore hard to detect among small numbers of subjects. There are differences between pregnancy outcomes from embryos transferred during ovarian stimulation cycles and those from cryopreserved embryos transferred in a non-stimulated cycle (Hann *et al.* 2018), with some evidence that this is driven by endometrial effects (Conrad 2020), but improved study design is needed to elucidate the mechanisms.

Pathologies not directly relating to fertility were often well defined using established criteria that should help standardise participant groups; however, reviewing studies of eutopic endometrial transcriptomes in endometriosis we found almost no consistency in reported DEG. Aside from very low study power, differences in endometriotic grade inclusion criteria could be responsible for this variation (Poli-Neto *et al.* 2020). In contrast, RIF was inconsistently defined between studies, but these studies contained the largest number of participants in our comparisons. Still, only one DEG was consistently reported in >2 studies comparing RIF to controls, likely also reflecting participant variability. No definition of RIF has been agreed, although defective endometrial receptivity is proposed as a key component in the condition especially in light of embryo and endometrial selection technology through pre-implantation genetic testing and transfer in non-stimulated cycles (Santamaria & Simon 2021). However, there is a growing clamour to characterise the condition as iatrogenic with no underlying biological mechanism (Somigliana *et al.* 2018, Ben Rafael 2020), and despite the limitations of DEG comparison between studies, our analysis supports this contention.

Common DEG were much more readily identified between studies characterising the transcriptome of the secretory phase, and these genes and associated biological processes reflect meta-analysis-validated gene networks underpinning this cycle phase (Altmae *et al.* 2017). However, *IL15* was the only consistently identified DEG, and this gene is an important regulator of natural killer cells; innate immune cells with cytotoxic and inflammatory roles are highly prevalent in the secretory phase endometrium (Fraser & Zenclussen 2022).* IL15* secreted by decidualising endometrial stromal cells has been characterised as an important recruitment and maturation factor for uNK cells (Kitaya *et al.* 2005, Murata *et al.* 2020) and is implicated in regulating the clearance of senescent decidual stromal cells by uNK (Brighton *et al.* 2017). Immune response pathways driven by increased expression of *IL15*, *C4BPA*, *CD55* and *SERPING1* were also prevalent in the mid-secretory endometrium in many studies, highlighting that inflammatory and immune recognition processes discriminate the mid-secretory phase and play a prominent role in embryo implantation (Robertson *et al.* 2022). In contrast, the lack of any functional annotations associated with similar numbers of cDEG from studies investigating ovarian stimulation and RIF suggests that biological mechanisms underpinning differences between these groups cannot be determined when group definitions are heterogeneous, and perhaps that differential expression analysis in bulk tissue samples is not able to resolve these mechanisms.

New analyses using network biology approaches are available to identify causal gene networks within and outside of DEG, and applying these to the endometrium, coupled with insights from single-cell transcriptomics, may offer better characterisation of gene function (Aplin & Stevens 2022). Transcriptomics is a powerful tool for studying biology and disease aetiology, and future transcriptomic studies of the endometrium should not focus on reporting DEG identified in low power studies but instead design comparisons, apply network techniques to enrich for causal genes and pathways, and if possible follow up with in vitro experiments that offer stringent variable control. Sophisticated endometrial organoid systems are now established, enabling interactions between different cell types inferred from transcriptome studies to be functionally investigated (Rawlings *et al.* 2021). Moreover, *in vitro* embryonic development within the endometrial niche is now accessible with the advent of stem cell-derived embryo-like structures (Kagawa *et al.* 2022).

## Supplementary Materials

Table S1. 74 studies that met the inclusion criteria of the literature search, and the associated metadata.

Table S2. Commonly reported differentially expressed genes common to ≥3 studies in studies comparing secretory vs proliferative endometrium, and their average fold change in expression.

Table S3. Commonly reported differentially expressed genes common to ≥4 studies in studies comparing mid-secretory vs early secretory endometrium, and their average fold change in expression.

Table S4. Commonly reported differentially expressed genes common to ≥2 studies in studies comparing mid-secretory endometrium from ovarian stimulation-treated participants vs controls and their average fold change in expression, and their average fold change in expression.

Table S5. Commonly reported differentially expressed genes common to ≥2 studies in studies comparing mid-secretory endometrium from RIF patients vs controls, and their average fold change in expression.

## Declaration of interest

The authors declare that there is no conflict of interest that could be perceived as prejudicing the impartiality of the research reported..

## Funding

This work was supported by funds from Department of Health Scientist Practitioner Training Scheme, and an NIHR Pre-doctoral Clinical Academic Fellowship.

## Author contribution statement

PTR and DRB designed the project. ERW and MM performed the systematic review, and ERW and PTR analysed the included studies. PTR, ERW and JDA wrote the manuscript, which was edited by DRB.
