# Supplementary material for: A systematic review of transcriptomic studies of the human endometrium reveals inconsistently reported differentially expressed genes
Source: Reprod Fertil. 2023 Jul 7;4(3):e220115. doi: 10.1530/RAF-22-0115 (PMC10388686; doi:10.1530/RAF-22-0115)
Supplement: Table S1. 74 studies that met the inclusion criteria of the literature search, and the associated metadata. [file supplementary_table_1.pdf]

Supplementary Table 1

First Author	Date	Investigative category	Study Location	Number of participants	Age	Ethnicity	BMI	Menstrual Cycle Length	Parity	Day(s) of collection	Endometrial location of sampling	Endometrial thickness	Sampling	Pathology (Classification)	Hormonal treatment	RNA Method	Sequencing platform	Raw Data Availability
Carson	2002	Across menstrual cycle	USA	6						LH2-4 & LH7-9				Fertile		microarray	Affymetrix GeneChip HU_U95	
Kao	2002	Across menstrual cycle	USA	11	28-39			26-35		LH8-10	fundus		Pipelle	None		microarray	Affymetrix GeneChip Hu95A	
Borthwick	2003	Across menstrual cycle	UK	10	23-44 (35.7)			28-32		d9-11 + LH6-8				Infertility (various)		microarray	Affymetrix GeneChip HG_U95 (A-E)	
Riesewijk	2003	Across menstrual cycle	Spain	8	23-39	Caucasian		normal		LH2 &LH 7			Pipelle	None		microarray	Affymetrix GeneChip Hu95A	
Kao	2003	Pathology	USA	8	28-39			26-35		LH8-10	fundus		Pipelle	Endometriosis		microarray	Affymetrix GeneChip Hu95A	
Ponnampalam	2004	Across menstrual cycle	Australia	43	18-47			normal		histological stages across cycle				None		microarray	Peter MacCallum Cancer Institute Array	
Mirkin	2004	Hormone treatment; across menstrual cycle	USA	15	24-32			regular		d21	fundus		Pipelle	Fertile	natural cycle + Follistim+ Ganirelix/ GonalF+C ertrorelix	microarray	Affymetrix GeneChip Hu95A	
Horcajadas	2005	Hormone treatment; across menstrual cycle	Spain	14	23-39	Caucasion				hCG+7	fundus		Pipelle	fertile	leuprolide acetate + FSH + HMG + hCG	microarray	Affymetrix GeneChip HG_U133A	
Mirkin	2005	Across menstrual cycle	Canada	8	18-35		18-29	normal		LH3 & LH8	fundus		Pipelle	None		microarray	Affymetrix GeneChip Hu95Av2	
Simon	2005	Hormone treatment	Spain	12	24.6-26.0		21.1-23.1	24-35		hCG+7	fundus		Pipelle	None	Natural + Ganirelix + high-dose ganirelix + buseralin	microarray	Affymetrix GeneChip HG_U133A	
Critchley	2006	Pathology	UK	10	30-48			25-35		histological mid- and late-secretory				Heavy bleeding		microarray	Affymetrix GeneChip HG_U133A	GPX-000001
Talbi	2006	Across menstrual cycle	USA	28	23-50	Caucasion, Black, Asian, Other		24-35		histological stages across cycle			Pipelle or Curette	None + fibroids +pelvic pain + ovarian cyst		microarray	Affymetrix GeneChip HG_U133A Plus 2.0	GSE4888
Burney	2007	Pathology; across menstrual cycle	USA	21	22-44	Caucasion, Black, Asian, Asian-Indian, Other		normal		histological stages across cycle	fundus		Pipelle or Curette	Endometriosis (American Fertility Classification)		microarray	Affymetrix GeneChip HG_U133A Plus 2.0	GSE6364
Otsuka	2007	Across menstrual cycle	Brazil	24	47.7					histological proliferative and mid-secretory				Fibroids		microarray	CodeLink UniSet Human I Bioarray	
Borghese	2008	Pathology	France	12		Caucasion				histological secretory				Endometriosis (stage IV) (American Fertility Classification)		microarray	NimbleGen Array	GSE12768
Horcajadas	2008	Hormone treatment; across menstrual cycle	Spaain	50	23-39		19-25			hCG+1,+3, +5,+7,+9	fundus		Pipelle	None	leuprolide acetate + FSH + HMG + hCG	microarray	Affymetrix GeneChip HG_U133A	
Liu	2008	Hormone treatment	China	13	26-38			regular		hCG+7	fundal and upper uterus		Pipelle	Infertile	Buseralin + HMG +hCG	microarray	Affymetrix GeneChip HG_U133A	
Macklon	2008	Hormone treatment	Netherlands	8	31-38			25-35		ovulation(scan)+5	fundus		Pipelle	None	recFSH + Orgalutran +hCG	microarray	Affymetrix GeneChip HG_U133A Plus 2.0	

Sherwin	2008	Pathology	Belgium	14				25-32		histological stages across cycle& d23-26			Pipelle	Endometriosis (American Fertility Classification)		microarray	Custom Array	
Tapia	2008	Pathology; hormone treatment	Chile	17	26-47		22.6-29.4			day 20 from estrogen start	fundus		Pipelle	Implantation Failure (>1 failed oocyte donation cycle)	Estrogen and Progester one	microarray	Incyte's human UniGEM 2.0 library	
Van Vaerenbergh	2009	Hormone treatment; Across menstrual cycle	Belgium	11	<37					hCG+36hrs			Pipelle	none	rec-FSH (Puregon) + GNRH Antagonist (Orgalutra n) + hCG (Pregnyl)	microarray	Affymetrix GeneChip HG_U133A Plus 2.0	GSE13027
Haouzi	2009a	Across menstrual cycle	France	31	30.4					LH2 & LH7				Male Factor		microarray	Affymetrix GeneChip HG_U133A Plus 2.0	
Haouzi	2009b	Hormone treatment; across menstrual cycle	France	21	30.9					LH2 & LH7, hCG+2 & hCG+5				Male factor		microarray	Affymetrix GeneChip HG_U133A Plus 2.0	
Koler	2009	Pathology	Israel	22	19-40					d21		10.5±1.9	Pipelle	RIF (at least 3 failed ET with 10 good quality embryos)		microarray	Array-Ready Oligo SetTM for the Human Genome Version 3.0	
Van Vaerenbergh	2010	Across menstrual cycle	Belgium	1	24			regular	2	LH6			Pipelle	Male factor		microarray	Affymetrix GeneChip HG_U133A Plus 2.0	
Altmae	2010	Pathology	Sweden	9	31.8 and 30.5		23.5 and 22.7	28.4 and 28.2	1.5±0.2	LH7	anterior wall	9.8±1.2 (mid-secretory)	Pipelle	Unexplained Infertility		microarray	Agilent Whole Human Genome Oligo Microarray	GSE16532
Tseng	2010	Across menstrual cycle	China	28	23-50			24-35		histological stages across cycle				fibroids, prolapse, pelvic pain		microarray	Affymetrix GeneChip HG_U133A Plus 2.0	GDS4888
Kuokkanen	2010	Across menstrual cycle	USA	8	24-29		21.5-24.3	25-35		d12 & d20-23			Pipelle	None		microarray	Affymetrix GeneChip HG_U133A Plus 2.0	
Haouzi	2010	Hormone treatment; across menstrual cycle	France	14	27.4		24			hCG+2 & hCG+5				Male factor	antagonist	microarray	Affymetrix GeneChip HG_U133A Plus 2.0	
Labarta	2011	Hormone treatment	Spain	12	27.5 and 22		22.4 and 22.8	25-35	3/6 and 1/6	hCG+7	fundus		Pipelle	None	Procrin or Cetrotide then Gonal-F then Ovitrelle	microarray	Agilent Whole Human Genome Oligo Microarray	
Blockeel	2011	Hormone treatment	Belgium	10	30.4		23.3 and 22.1			hCG+36hrs			Pipelle	Infertile	GnCR antagonist recFSH (Puregon) or recFSH then hCG (Pregnyl)	microarray	Affymetrix GeneChip HG_U133A Plus 2.0	GSE18557
Van Vaerenbergh	2011	Hormone treatment	Belgium	14						hCG+36hrs			Pipelle	Infertility	rec-FSH (Puregon) + GNRH Antagonist (Orgalutra n) + hCG (Pregnyl) + Utrogesta n	microarray	Affymetrix GeneChip HG_U133A Plus 2.0	GSE19959
Hawkins	2011	Pathology; across menstrual cycle	USA	19	34 and 45	Latina, african-american, caucasian		regular		histological stages across cycle			Curettage	Endometriosis		microarray	Illumina Human WG-6 version 2.0 BeadChips	GSE23339

Ledee	2011	Pathology	France	15	25-37		19-29			ovulation+7-9			Pipelle	implantation failure (>10 good quality embryos transferred) and RM (3 unexplained 6-12 week miscarriages)		microarray	Affymetrix GeneChip HG_U133A Plus 2.0	GSE26787
Haouzi	2011	Hormone treatment	France	18	<36					hCG+5				Male factor	long agonist or antagonist	microarray	Affymetrix GeneChip HG_U133A Plus 2.0	
Aghajanova	2011	Pathology; across menstrual cycle	USA	63	35.7 and 32.2	Caucasian, Black, Mixed, Asian, Other				histological stages across cycle			Biopsy and hysterectomy	Endometriosis (Revised American Fertility Classification)		microarray	Affymetrix GeneChip HG_U133A Plus 2.0	
Bellver	2011	Pathology; hormone treatment	Spain	28	18-35		22-38	25-35 and PCOS (>35 days and oligoanovulation)		hCG+7				PCOS (Rotterdam Criteria)	recFSH + hCG	microarray	Affymetrix GeneChip HG_U133A	
Altmae	2012	Across menstrual cycle	Sweden	8	34±10		25.7±7.2	27.6±1.1	2.4±1.5	d7	anterior wall		Pipelle	None		microarray	Affymetrix GeneChip HG_U133A Plus 2.0	E-MEXP-2359 (Array Express
Petracco	2012	Across menstrual cycle	USA	9	36+34.7+37.0					d1-3, d5-8, d11-13			Pipelle	none		microarray	Affymetrix GeneChip Human Gene 1.0 ST	
Othman	2012	Pathology	Malaysia	17	26-55					d21-24				RM (loss of 2 or more before 20wks)		microarray	Affymetrix GeneChip Human ST	
Hu	2014	Across menstrual cycle	China	6	24-34			normal		LH2 & LH6	fundus		Pipelle	None		RNA-Seq	Illumina	
Haouzi	2014	Hormone treatment; across menstrual cycle	France	15	31±4.9 + 30.1±2.7					hCG+2 & hCG+5				male factor	Agonist and Antagonist	microarray	Affymetrix GeneChip HG_U133A Plus 2.0	
Zieba	2015	Across menstrual cycle	Sweden	5	34-83								Hysterectomy samples	polyp/fibroids/endometrial carcinoma		RNA-Seq	Illumina HiSeq2000 and 25000	E-MTAB-1722
Kosova	2015	Pathology	USA	37	33.4-36.3	European Ancestry				LH9-11				Recurrent early pregnancy loss (2 or more unexplained misc. at <10wks)		microarray	Illumina HT-12 v4 BeadChip	GSE63901
Cuevas	2016	Hormone treatment; across menstrual cycle	Chile	14	36.1 + 35.4	Hispanic	25.2 and 25.6	26-35	~3.3 and 3.0	LH2 & LH7	fundus		Pipelle	None	Mifepristone	microarray	Affymetrix GeneChip HG_U133A	
Koot	2016	Pathology	Netherlands	115	34.0 + 34.6		19-53	25-35	≤3	LH6/7			Endobiops Standard CH9	RIF (≥3 failed IVF/ICSI or ≥10 failed ET)		microarray	Agilent 4x44k	GSE58144 and E-MTAB-2591
Altmae	2016	Pathology; hormone treatment	Spain	15	30.2±4.3 + 32.4±5 + 31.8±3.8		20.7-23.5	28.2	1.5 (fertile controls)	LH7 & P+6	anterior wall	9.3±0.9 + 8.5+1.7	Pipelle	RIF (2-4 failed ET)	Progynon + Progesterone	microarray	Agilent Whole Human Genome Oligo Microarray	E-MTAB-3713
Herndon	2016	Pathology	USA	8	39.6±1.3 + 36.6±1.6	Black, White, Asian, Hispanic			2-5	histological proliferative			Pipelle and hysterectomy	Adenomyosis (histology)		microarray	Affymetrix Human Gene 1.0 ST	GSE78851
Jiang	2016	Pathology	China	8	44.4			25-35		histological proliferative			hysterectomy	Adenomyosis (histology)		microarray	Affymetrix Human Transcriptome Array 2.0	GSE68870
Choi	2016	Pathology	South Korea	10	35.13 + 32.85			normal		d21			pipelle	RIF (3 or more cycles with good quality embryos)		microarray	Agilent Human Genome 8x60K	
Aghajanova	2017	Pathology; across menstrual	USA	48	23-50	Black, Caucasian, Asian,				histological stages across			Pipelle and hysterectomy	Fibroids		microarray	Affymetrix Human Gene 1.0 ST	GSE51981

		cycle				Unknown				cycle								
Huang	2017	Pathology	China	14	36.6 + 35.3 + 31.8		21.8 + 20.6 + 21.1	29.1+28.5+ 30.0		LH7		~10.2+9.8+10.5	Pipelle	RIF (ET of 4 good quality embryos in at least 3 cycles)+ RM (3 or more consecutive miscarriages under 24 weeks)		RNA-Seq	Illumina HiSeq2000	PRJNA314429 (NCBI)
Young	2017	Hormone treatment	USA	47	27			25-35		LH10			Pipelle	None	Leuprolide acetetae + Estradiol + Progesterone (Varying)	microarray	Illumina HT-12 v4 BeadChip	GSE56980
Sigurgeirsson	2017	Across menstrual cycle	Sweden	7	24-31			regular		d6-8 & LH7-9			Curette	none		RNA-Seq	Illumina HiSeq 2500	GSE86491
Chen	2017	Pathology	Australia	6			35.3			?			Hysterectomy	endometrial cancer (FIGO)		RNA-Seq	Illumina HiSeq 2500	
Fung	2017	Across menstrual cycle	Australia	123	31.5± 0.7	European Ancestry				histological stages across cycle			curettage	Endometriosis, fibroids, adenomyosis		microarray	Illumina HT-12 v4 BeadChip	
Rahmioglu	2017	Pathology; across menstrual cycle	UK	16				regular (predictable within one week)		self reported stages across cycle				Endometriosis		microarray	Illumina HT-12 v4 BeadChip	
Cui	2018	Pathology	China	10	26-41 + 23-40			regular		proliferative				Endometriosis (American Fertility Classification)		RNA-Seq	Illumina HiSeq 2500	
Fung	2018	Pathology; across menstrual cycle	Australia	229	31.21 ±0.53 and 36.56 ±0.51	European Ancestry				histological stages across cycle			curettage	Endometriosis, fibroids, adenomyosis		microarray	Illumina HT-12 v4 BeadChip	
Suhortshenko	2018	Across menstrual cycle	Estonia and Spain	35	29.1 + 30.2		23.2	regular	≥1	LH1-3 & LH7-9			Pipelle	fertile		RNA-Seq	Illumina HiSeq2000 and 25000	GSE98386
Bastu	2019	Pathology	Turkey	48	31.3 + 32.76		23.15 + 25.36		1 or more (controls)	LH7-10			Pipelle	RIF (failure in 3 or more cycles with 1 or more good quality in each)		microarray	Agilent Sure Print G3 8x60K	GSE111974
Chen	2019	Pathology	China	6				28-35		LH3-7		>4mm		RIF (3 fresh or frozen ET with grade 2 or above (Dale scoring))		RNA-Seq	Illumina HiSeq X Ten	
Cheng	2019	Pathology	China	12	31+27.5		21.52 +20.77	58.7+32.08		mid-luteal				PCOS, low reserve, salpingitis		RNA-Seq	Illumina HiSeq 2500	
Li	2020	Hormone treatment; across menstrual cycle	China	12	32.5+ 31+34.3		21.2+ 19.8+ 20.6	31.75+27.5 +29.0		d11-14, LH7 & hCG+7	fundus		Pipelle	Tubal	Long agonist(Decapeptyl +GonalF+ Triptorelin) Antagonist (Cetrotide +hCG)	RNA-Seq	Illumina HiSeq x Ten	Supplementary to paper
Crosby	2020	Pathology	Ireland	20	35.3+ 35.2		23.7+ 22.8	29.8+28.8		LH7		~8.8+10.1	Pipelle	Unexplained (Zegers-Hochschild 2017)		RNA-Seq	Illumina NextSeq 500	GSE144895
Osman	2020	Hormone treatment	USA	14	30.5± 6.45		28.3± 3.85			d16			Pipelle	None	leuprolide acetate + Estradiol Valerate	RNA-Seq	Illumina HiSeq 2500	
Barrios-Hernández	2020	Hormone treatment	Mexico	10	22-35 years (29 ± 4.5)			regular 24-37 days (28.5 ± 3.95)		6 days post-follicle rupture (ultrasaound)	Posterior wall of the uterine cavity			None	Levonorgestrel	microarray	Affymetrix GeneChip Human Genome U133 Plus 2.0	
Mortlock	2020	Pathology	Australia	206		European Ancestry				histological stages across			curettage	Endometriosis		RNA-seq	Illumina HiSeq 4000	http://reproductivegenomics.com.au/shin

										cycle								y/endo_eqtl_r na/
Joshi	2021	Pathology	USA	11	18–45 years			regular		mid-secretory				Endometriosis (not defined)		microarray	Affymetrix Human Gene 1.0 ST Array	Dryad Digital Repository
Adamyan	2021	Pathology	Russia	33	32-38 years			regular						Endometriosis (ASRM classification stage III-IV)		RNA-Seq	Illumina HiSeq 3000	
Suthaporn	2021	Across menstrual cycle	UK	12	18–35 years	Caucasian	18-25	regular 25–35 days		day LH+7 to LH+9	All four walls of endometrial cavity		Pipelle	None		microarray	Affymetrix Clariom S Human Array	GSE143620
Pierzyński	2021	Hormone treatment	Poland	45	18-37	Asian, Black or African American, Caucasian, North American, South American, European descent, Other	18-30	healthy patients?		24 hours post-nolasiban or placebo			Pipelle	None	Estrogen and Progester one	RNA-Seq	Illumina NextSeq 500	
Salamun	2021	Pathology	Italy	14	<38 years		25.9-39.3			day 21-23 of menstrual cycle			Rampipell a catheter	unexplained infertility		RNA-Seq	Illumina Hiseq 2000	
Prašnikar	2022	Pathology	Slovenia	20	≤ 42 years		17.3-34.6	regular (24 – 36 days)		LH+7 to LH+9		4.6-11.2	Pipelle	Adenomyosis (sonography)		RNA-Seq	Illumina NovaSeq 6000	GSE185392

References

Carson DD, Lagow E, Thathiah A, Al-Shami R, Farach-Carson MC, Vernon M, Yuan L, Fritz MA & Lessey B 2002 Changes in gene expression during the early to mid-luteal (receptive phase) transition in human endometrium detected by high-density microarray screening. Molecular Human Reproduction 8 871–879. (https://doi.org/10.1093/molehr/8.9.871)

Kao LC, Tulac S, Lobo S, Imani B, Yang JP, Germeyer A, Osteen K, Taylor RN, Lessey BA & Giudice LC 2002 Global gene profiling in human endometrium during the window of implantation. Endocrinology 143 2119–2138. (https://doi.org/10.1210/endo.143.6.8885)

Borthwick JM, Charnock-Jones DS, Tom BD, Hull ML, Teirney R, Phillips SC & Smith SK 2003 Determination of the transcript profile of human endometrium. Molecular Human Reproduction 9 19–33. (https://doi.org/10.1093/molehr/gag004)

Riesewijk A, Martín J, van Os R, Horcajadas JA, Polman J, Pellicer A, Mosselman S & Simón C 2003 Gene expression profiling of human endometrial receptivity on days LH+2 versus LH+7 by microarray technology. Molecular Human Reproduction 9 253–264. (https://doi.org/10.1093/molehr/gag037)

Kao LC, Germeyer A, Tula S, Lobo S, Yang JP, Taylor RN, Osteen K, Lessey BA & Giudice LC 2003 Expression profiling of endometrium from women with endometriosis reveals candidate genes for disease-based implantation failure and infertility. Endocrinology 144 2870–2881. (https://doi.org/10.1210/en.2003-0043)

Ponnampalam AP, Weston GC, Trajstman AC, Susil B & Rogers PAW 2004 Molecular classification of human endometrial cycle stages by transcriptional profiling. Molecular Human Reproduction 10 879–893. (https://doi.org/10.1093/molehr/gah121)

Mirkin S, Nikas G, Hsiu JG, Díaz J & Oehninger S 2004 Gene expression profiles and structural/ functional features of the periimplantation endometrium in natural and gonadotropin-stimulated cycles. Journal of Clinical Endocrinology and Metabolism 89 5742–5752. (https://doi.org/10.1210/jc.2004-0605)

Horcajadas JA, Riesewijk A, Polman J, van Os R, Pellicer A, Mosselman S & Simón C 2005 Effect of controlled ovarian hyperstimulation in IVF on endometrial gene expression profiles. Molecular Human Reproduction 11 195–205. (https://doi.org/10.1093/molehr/gah150)

Mirkin S, Arslan M, Churikov D, Corica A, Diaz JI, Williams S, Bocca S & Oehninger S 2005 In search of candidate genes critically expressed in the human endometrium during the window of implantation. Human Reproduction 20 2104–2117. (https://doi.org/10.1093/humrep/dei051)

Simon C, Oberyé J, Bellver J, Vidal C, Bosch E, Horcajadas JA, Murphy C, Adams S, Riesewijk A, Mannaerts B, et al. 2005 Similar endometrial development in oocyte donors treated with either high- or standard-dose GnRH antagonist compared to treatment with a GnRH agonist or in natural cycles. Human Reproduction 20 3318–3327. (https://doi.org/10.1093/humrep/dei243)

Critchley HOD, Robertson KA, Forster T, Henderson TA, Williams ARW & Ghazal P 2006 Gene expression profiling of mid to late secretory phase endometrial biopsies from women with menstrual complaint. American Journal of Obstetrics and Gynecology 195 406.e1–406.16. (https://doi.org/10.1016/j.ajog.2006.05.002)

Talbi S, Hamilton AE, Vo KC, Tulac S, Overgaard MT, Dosiou C, Le Shay N, Nezhat CN, Kempson R, Lessey BA, et al. 2006 Molecular phenotyping of human endometrium distinguishes menstrual cycle phases and underlying biological processes in normo-ovulatory women. Endocrinology 147 1097–1121. (https://doi.org/10.1210/en.2005-1076)

Burney RO, Talbi S, Hamilton AE, Vo KC, Nyegaard M, Nezhat CR, Lessey BA & Giudice LC 2007 Gene expression analysis of endometrium reveals progesterone resistance and candidate susceptibility genes in women with endometriosis. Endocrinology 148 3814–3826. (https://doi.org/10.1210/en.2006-1692)

Otsuka AY, Andrade PM, Villanova FE, Borra RC & Silva IDCG 2007 Human endometrium mRNA profile assessed by oligonucleotide three-dimensional microarray. Gynecological Endocrinology 23 527–534. (https://doi.org/10.1080/09513590701550221)

Borghese B, Mondon F, Noël JC, Fayt I, Mignot TM, Vaiman D & Chapron C 2008 Gene expression profile for ectopic versus eutopic endometrium provides new insights into endometriosis oncogenic potential. Molecular Endocrinology 22 2557–2562. (https://doi.org/10.1210/me.2008-0322)

Horcajadas JA, Mínguez P, Dopazo J, Esteban FJ, Domínguez F, Giudice LC, Pellicer A & Simón C 2008 Controlled ovarian stimulation induces a functional genomic delay of the endometrium with potential clinical implications. Journal of Clinical Endocrinology and Metabolism 93 4500–4510. (https://doi.org/10.1210/jc.2008-0588)

Liu Y, Lee KF, Ng EHY, Yeung WSB & Ho PC 2008 Gene expression profiling of human peri-implantation endometria between natural and stimulated cycles. Fertility and Sterility 90 2152–2164. (https://doi.org/10.1016/j.fertnstert.2007.10.020)

Macklon NS, van der Gaast MH, Hamilton A, Fauser BCJM & Giudice LC 2008 The impact of ovarian stimulation with recombinant FSH in combination with GnRH antagonist on the endometrial transcriptome in the window of implantation. Reproductive Sciences 15 357–365. (https://doi.org/10.1177/1933719107311781)

Sherwin JRA, Sharkey AM, Mihalyi A, Simsa P, Catalano RD & DHooghe TM 2008 Global gene analysis of late secretory phase, eutopic endometrium does not provide the basis for a minimally invasive test of endometriosis. Human Reproduction 23 1063–1068. (https://doi.org/10.1093/humrep/den078)

Tapia A, Gangi LM, Zegers-Hochschild F, Balmaceda J, Pommer R, Trejo L, Pacheco IM, Salvatierra AM, Henríquez S, Quezada M, et al. 2008 Differences in the endometrial transcript profile during the receptive period between women who were refractory to implantation and those who achieved pregnancy. Human Reproduction 23 340–351. (https://doi.org/10.1093/humrep/dem319)

Van Vaerenbergh I, Van Lommel L & Ghislain V 2009 In GnRH antagonist/rec-FSH stimulated cycles, advanced endometrial maturation on the day of oocyte retrieval correlates with altered gene expression. Human Reproduction 24 1085–1091. (https://doi.org/ 10.1093/humrep/den501)

Haouzi D, Assou S, Mahmoud K, Tondeur S, Rème T, Hedon B, De Vos J & Hamamah S 2009a Gene expression profile of human endometrial receptivity: comparison between natural and stimulated cycles for the same patients. Human Reproduction 24 1436–1445. (https://doi.org/10.1093/humrep/dep039)

Haouzi D, Mahmoud K, Fourar M, Bendhaou K, Dechaud H, De Vos J, Rème T, Dewailly D & Hamamah S 2009b Identification of new biomarkers of human endometrial receptivity in the natural cycle. Human Reproduction 24 198–205. (https://doi.org/10.1093/humrep/den360)

Koler M, Achache H, Tsafrir A, Smith Y, Revel A & Reich R 2009 Disrupted gene pattern in patients with repeated in vitro fertilization (IVF) failure. Human Reproduction 24 2541–2548. (https://doi.org/10.1093/humrep/dep193)

Van Vaerenbergh I, McIntire R, Van Lommel L, Devroey P, Giudice L & Bourgain C 2010 Gene expression during successful implantation in a natural cycle. Fertility and Sterility 93 268.e15–268. e18. (https://doi.org/10.1016/j.fertnstert.2009.08.057)

Altmäe S, Martínez-Conejero JA, Salumets A, Simón C, Horcajadas JA & Stavreus-Evers A 2010 Endometrial gene expression analysis at the time of embryo implantation in women with unexplained infertility. Molecular Human Reproduction 16 178–187. (https://doi.org/10.1093/molehr/gap102)

Tseng LH, Chen I, Chen MY, Yan H, Wang CN & Lee CL 2010 Genome-based expression profiling as a single standardized microarray platform for the diagnosis of endometrial disorder: an array of 126-gene model. Fertility and Sterility 94 114–119. (https://doi.org/10.1016/j.fertnstert.2009.01.130)

Kuokkanen S, Chen B, Ojalvo L, Benard L, Santoro N & Pollard JW 2010 Genomic profiling of microRNAs and messenger RNAs reveals hormonal regulation in microRNA expression in human endometrium. Biology of Reproduction 82 791–801. (https://doi.org/10.1095/biolreprod.109.081059)

Haouzi D, Assou S, Dechanet C, Anahory T, Dechaud H, De Vos J & Hamamah S 2010 Controlled ovarian hyperstimulation for in vitro fertilization alters endometrial receptivity in humans: protocol effects. Biology of Reproduction 82 679–686. (https://doi.org/10.1095/biolreprod.109.081299)

Labarta E, Martinez-Conejero JA, Alama P, Horcajadas JA, Pellicer A, Simon C & Bosch E 2011 Endometrial receptivity is affected in women with high circulating progesterone levels at the end of the follicular phase: a functional genomics analysis. Human Reproduction 26 1813–1825. (https://doi.org/10.1093/humrep/der126)

Blockeel C, Van Vaerenbergh I, Fatemi HM, Van Lommel L, Devroey P & Bourgain C 2011 Gene expression profile in the endometrium on the day of oocyte retrieval after ovarian stimulation with low-dose hCG in the follicular phase. Molecular Human Reproduction 17 33–41. (https://doi.org/10.1093/molehr/gaq070)

Van Vaerenbergh I, Fatemi HM, Blockeel C & Van Lommel L 2011 Progesterone rise on HCG day in GnRH antagonist/rFSH stimulated cycles affects endometrial gene expression. Reproductive Biomedicine Online 22 263–271. (https://doi.org/ 10.1016/j.rbmo.2010.11.002)

Hawkins SM, Creighton CJ, Han DY, Zariff A, Anderson ML, Gunaratne PH & Matzuk MM 2011 Functional microRNA involved in endometriosis. Molecular Endocrinology 25 821–832. (https://doi.org/10.1210/me.2010-0371)

Lédée N, Munaut C, Aubert J, Sérazin V, Rahmati M, Chaouat G, Sandra O & Foidart JM 2011 Specific and extensive endometrial deregulation is present before conception in IVF/ICSI repeated implantation failures (IF) or recurrent miscarriages. Journal of Pathology 225 554–564. (https://doi.org/10.1002/path.2948)

Haouzi D, Dechaud H, Assou S, Monzo C, de Vos J & Hamamah S 2011 Transcriptome analysis reveals dialogues between human trophectoderm and endometrial cells during the implantation period. Human Reproduction 26 1440–1449. (https://doi.org/10.1093/humrep/der075)

Aghajanova L & Giudice LC 2011 Molecular evidence for differences in endometrium in severe versus mild endometriosis. Reproductive Sciences 18 229–251. (https://doi.org/10.1177/1933719110386241)

Bellver J, Martínez-Conejero JA, Labarta E, Alamá P, Melo MAB, Remohí J, Pellicer A & Horcajadas JA 2011 Endometrial gene expression in the window of implantation is altered in obese women especially in association with polycystic ovary syndrome. Fertility and Sterility 95 2335. (https://doi.org/10.1016/j.fertnstert.2011.03.021)

Altmäe S, Reimand J, Hovatta O, Zhang P, Kere J, Laisk T, Saare M, Peters M, Vilo J, Stavreus-Evers A, et al. 2012 Research resource: interactome of human embryo implantation: identification of gene expression pathways, regulation, and integrated regulatory networks. Molecular Endocrinology 26 203–217. (https://doi.org/10.1210/me.2011-1196)

Petracco RG, Kong A, Grechukhina O, Krikun G & Taylor HS 2012 Global gene expression profiling of proliferative phase endometrium reveals distinct functional subdivisions. Reproductive Sciences 19 1138–1145. (https://doi.org/10.1177/1933719112443877)

Othman R, Omar MH, Shan LP, Shafiee MN, Jamal R & Mokhtar NM 2012 Microarray profiling of secretory-phase endometrium from patients with recurrent miscarriage. Reproductive Biology 12 183–199. (https://doi.org/10.1016/s1642-431x(12)60085-0)

Hu S, Yao G, Wang Y, Xu H, Ji X, He Y, Zhu Q, Chen Z & Sun Y 2014 Transcriptomic changes during the pre-receptive to receptive transition in human endometrium detected by RNA-Seq. Journal of Clinical Endocrinology and Metabolism 99 E2744–E2753. (https://doi.org/10.1210/jc.2014-2155)

Haouzi D, Bissonnette L, Gala A, Assou S, Entezami F, Perrochia H, Dechaud H, Hugues JN & Hamamah S 2014 Endometrial receptivity profile in patients with premature progesterone elevation on the day of hCG administration. BioMed Research International 2014 951937. (https://doi.org/10.1155/2014/951937)

Zieba A, Sjöstedt E, Olovsson M, Fagerberg L, Hallström BM, Oskarsson L, Edlund K, Tolf A, Uhlen M & Ponten F 2015 The human endometrium-specific proteome defined by transcriptomics and antibody-based profiling. Omics 19 659–668. (https://doi.org/10.1089/omi.2015.0115)

Kosova G, Stephenson MD, Lynch VJ & Ober C 2015 Evolutionary forward genomics reveals novel insights into the genes and pathways dysregulated in recurrent early pregnancy loss. Human Reproduction 30 519–529. (https://doi.org/10.1093/humrep/deu355)

Cuevas CA, Tapia-Pizarro A, Salvatierra AM, Munroe DJ, Velasquez L & Croxatto HB 2016 Effect of single post-ovulatory administration of mifepristone (RU486) on transcript profile during the receptive period in human endometrium. Reproduction 151 331–349. (https://doi.org/10.1530/REP-15-0458)

Koot YE, van Hooff SR, Boomsma CM, van Leenen D, Groot Koerkamp MJ, Goddijn M, Eijkemans MJ, Fauser BC, Holstege FC & Macklon NS 2016 An endometrial gene expression signature accurately predicts recurrent implantation failure after IVF. Scientific Reports 6 19411. (https://doi.org/10.1038/srep19411)

Altmäe S, Tamm-Rosenstein K, Esteban FJ, Simm J, Kolberg L, Peterson H, Metsis M, Haldre K, Horcajadas JA, Salumets A, et al. 2016 Endometrial transcriptome analysis indicates superiority of natural over artificial cycles in recurrent implantation failure patients undergoing frozen embryo transfer. Reproductive Biomedicine Online 32 597–613. (https://doi.org/10.1016/j.rbmo.2016.03.004)

Herndon CN, Aghajanova L, Balayan S, Erikson D, Barragan F, Goldfien G, Vo KC, Hawkins S & Giudice LC 2016 Global transcriptome abnormalities of the eutopic endometrium from women with adenomyosis. Reproductive Sciences 23 1289–1303. (https://doi.org/10.1177/1933719116650758)

Jiang JF, Sun AJ, Xue W, Deng Y & Wang YF 2016 Aberrantly expressed long noncoding RNAs in the eutopic endometria of patients with uterine adenomyosis. European Journal of Obstetrics, Gynecology, and Reproductive Biology 199 32–37. (https://doi.org/10.1016/j.ejogrb.2016.01.033)

Choi Y, Kim HR, Lim EJ, Park M, Yoon JA, Kim YS, Kim EK, Shin JE, Kim JH, Kwon H, et al. 2016 Integrative analyses of uterine transcriptome and MicroRNAome reveal compromised LIFSTAT3 signaling and progesterone response in the endometrium of patients with recurrent/repeated implantation failure (RIF). PLoS One 11 e0157696. (https://doi.org/10.1371/journal.pone.0157696)

Aghajanova L, Houshdaran S, Irwin JC & Giudice LC 2017 Effects of noncavity-distorting fibroids on endometrial gene expression and function. Biology of Reproduction 97 564–576. (https://doi.org/10.1093/biolre/iox107)

Huang J, Qin H, Yang Y, Chen X, Zhang J, Laird S, Wang CC, Chan TF & Li TC 2017 A comparison of transcriptomic profiles in endometrium during window of implantation between women with unexplained recurrent implantation failure and recurrent miscarriage. Reproduction 153 749–758. (https://doi.org/10.1530/REP-16-0574)

Young SL, Savaris RF, Lessey BA, Sharkey AM, Balthazar U, Zaino RJ, Sherwin RA & Fritz MA 2017 Effect of randomized serum progesterone concentration on secretory endometrial histologic development and gene expression. Human Reproduction 32 1903–1914. (https://doi.org/10.1093/humrep/dex252)

Sigurgeirsson B, Åmark H, Jemt A, Ujvari D, Westgren M, Lundeberg J & Gidlöf S 2017 Comprehensive RNA sequencing of healthy human endometrium at two time points of the menstrual cycle. Biology of Reproduction 96 24–33. (https://doi.org/10.1095/biolreprod.116.142547)

Chen BJ, Byrne FL, Takenaka K, Modesitt SC, Olzomer EM, Mills JD, Farrell R, Hoehn KL & Janitz M 2017 Transcriptome landscape of long intergenic non-coding RNAs in endometrial cancer. Gynecologic Oncology 147 654–662. (https://doi.org/10.1016/j.ygyno.2017.10.006)

Fung JN, Girling JE, Lukowski SW, Sapkota Y, Wallace L, Holdsworth-Carson SJ, Henders AK, Healey M, Rogers PAW, Powell JE, et al. 2017 The genetic regulation of transcription in human endometrial tissue. Human Reproduction 32 893–904. (https://doi.org/10.1093/humrep/dex006)

Rahmioglu N, Drong AW, Lockstone H, Tapmeier T, Hellner K, Saare M, Laisk-Podar T, Dew C, Tough E, Nicholson G, et al. 2017 Variability of genome-wide DNA methylation and mRNA expression profiles in reproductive and endocrine disease related tissues. Epigenetics 12 897–908. (https://doi.org/10.1080/15592294.2017.1367475)

Cui D, Ma J, Liu Y, Lin K, Jiang X, Qu Y, Lin J & Xu K 2018 Analysis of long non-coding RNA expression profiles using RNA sequencing in ovarian endometriosis. Gene 673 140–148. (https://doi.org/10.1016/j.gene.2018.06.046)

Fung JN, Mortlock S, Girling JE, Holdsworth-Carson SJ, Teh WT, Zhu Z, Lukowski SW, McKinnon BD, McRae A, Yang J, et al. 2018 Genetic regulation of disease risk and endometrial gene expression highlights potential target genes for endometriosis and polycystic ovarian syndrome. Scientific Reports 8 11424. (https://doi.org/10.1038/s41598-018-29462-y)

Suhorutshenko M, Kukushkina V, Velthut-Meikas A, Altmäe S, Peters M, Mägi R, Krjutškov K, Koel M, Codoñer FM, Martinez-Blanch JF, et al. 2018 Endometrial receptivity revisited: endometrial transcriptome adjusted for tissue cellular heterogeneity. Human Reproduction 33 2074–2086. (https://doi.org/10.1093/humrep/dey301)

Bastu E, Demiral I, Gunel T, Ulgen E, Gumusoglu E, Hosseini MK, Sezerman U, Buyru F & Yeh J 2019 Potential marker pathways in the endometrium that may cause recurrent implantation failure. Reproductive Sciences 26 879–890. (https://doi.org/10.1177/1933719118792104)

Chen MY, Liao GD, Zhou B, Kang LN, He YM & Li SW 2019 Genomewide profiling of long noncoding RNA expression patterns in women with repeated implantation failure by RNA sequencing. Reproductive Sciences 26 18–25. (https://doi.org/10.1177/1933719118756752)

Cheng J, Jin X, Shen J, Mu Y, Li Q, Xia L, Gao Y & Xia Y 2019 Whole transcriptome sequencing reveals how acupuncture and moxibustion increase pregnancy rate in patients undergoing in vitro fertilizationembryo transplantation. BioMed Research International 2019 4179617. (https://doi.org/10.1155/2019/4179617)

Li L, Wang P, Liu S, Bai X, Zou B & Li Y 2020 Transcriptome sequencing of endometrium revealed alterations in mRNAs and lncRNAs after ovarian stimulation. Journal of Assisted Reproduction and Genetics 37 21–32. (https://doi.org/10.1007/s10815-019-01616-5)

Crosby DA, Glover LE, Brennan EP, Kelly P, Cormican P, Moran B, Giangrazi F, Downey P, Mooney EE, Loftus BJ, et al. 2020 Dysregulation of the interleukin-17A pathway in endometrial tissue from women with unexplained infertility affects pregnancy outcome following assisted reproductive treatment. Human Reproduction 35 1875–1888. (https://doi.org/10.1093/humrep/deaa111)

Osman EK, Wang T, Zhan Y, Juneau CR, Morin SJ, Seli E, Scott RT & Franasiak JM 2020 Varying levels of serum estradiol do not alter the timing of the early endometrial secretory transformation. Human Reproduction 35 1637–1647. (https://doi.org/10.1093/humrep/deaa135)

Barrios-Hernández AE, Durand-Carbajal M, Vega CC & Larrea F 2020 Analysis of the endometrial transcriptome at the time of implantation in women receiving a single post-ovulatory dose of levonorgestrel or mifepristone. Revista de Investigacion Clinica; Organo Del Hospital de Enfermedades de la Nutricion 72 363–371. (https://doi.org/10.24875/RIC.20000079)

Mortlock S, Kendarsari RI, Fung JN, Gibson G, Yang F, Restuadi R, Girling JE, Holdsworth-Carson SJ, Teh WT, Lukowski SW, et al. 2020 Tissue specific regulation of transcription in endometrium and association with disease. Human Reproduction 35 377–393. (https://doi.org/10.1093/humrep/dez279)

Joshi NR, Kohan-Ghadr HR, Roqueiro DS, Yoo JY, Fru K, Hestermann E, Yuan L, Ho SM, Jeong JW, Young SL, et al. 2021 Genetic and epigenetic changes in the utopic endometrium of women with endometriosis: association with decreased endometrial αvβ3 integrin expression. Molecular Human Reproduction 27 gaab018. (https://doi.org/10.1093/molehr/gaab018)

Adamyan L, Aznaurova Y, Stepanian A, Nikitin D, Garazha A, Suntsova M, Sorokin M & Buzdin A 2021 Gene expression signature of endometrial samples from women with and without endometriosis. Journal of Minimally Invasive Gynecology 28 1774–1785. (https://doi.org/10.1016/j.jmig.2021.03.011)

Suthaporn S, Jayaprakasan K, Thornton J, Walker K, Medrano JH, Castellanos M, May S, Polanski L, Raine-Fenning N & Maalouf WE 2021 Suboptimal mid-luteal progesterone concentrations are associated with aberrant endometrial gene expression, potentially resulting in implantation failure. Reproductive Biomedicine Online 42 595–608. (https://doi.org/10.1016/j.rbmo.2020.10.018)

Pierzyński P, Pohl O, Marchand L, Mackens S, Lorch U, Gotteland JP & Blockeel C 2021 The mechanism of action of oxytocin antagonist nolasiban in ART in healthy female volunteers. Reproductive Biomedicine Online 43 184–192. (https://doi.org/10.1016/j.rbmo.2021.01.003)

Salamun V, Bokal EV, Maver A & Papler TB 2021 Transcriptome study of receptive endometrium in overweight and obese women shows important expression differences in immune response and inflammatory pathways in women who do not conceive. PLoS One 16 e0261873. (https://doi.org/10.1371/journal.pone.0261873)

Prašnikar E, Kunej T, Gorenjak M, Potočnik U, Kovačič B & Knez J 2022 Transcriptomics of receptive endometrium in women with sonographic features of adenomyosis. Reproductive Biology and Endocrinology 20 2. (https://doi.org/10.1186/s12958-021-00871-5)
