# Supplementary material for: A systematic review of transcriptomic studies of the human endometrium reveals inconsistently reported differentially expressed genes
Source: Reprod Fertil. 2023 Jul 7;4(3):e220115. doi: 10.1530/RAF-22-0115 (PMC10388686; doi:10.1530/RAF-22-0115)
Supplement: Table S2. Commonly reported differentially expressed genes common to ≥3 studies in studies comparing secretory vs proliferative endometrium, and their average fold change in expression. [file supplementary_table_2.pdf]

Table S2

Gene Name	Number of studies reporting DEG	Average fold change (log_2)
APOD	4	3.65
ALDH1A3	4	3.62
MAOA	4	3.08
GABRP	4	2.00
OLFM1	4	-1.70
TNC	4	-2.41
SFRP1	4	-3.03
MT1G	3	4.55
CLDN4	3	4.22
DKK1	3	4.18
CYP26A1	3	3.86
SLC1A1	3	3.72
SCGB2A2	3	3.65
SPP1	3	3.45
MT1F	3	3.30
SGK1	3	3.20
TAP1	3	3.13
CFD	3	3.10
ARG2	3	3.08
C4BPA	3	3.00
SLC16A3	3	2.94
SCGB2A1	3	2.73
MAP3K5	3	2.24
MVP	3	2.12
MUC1	3	2.02
IL15	3	2.01
ID4	3	1.99
DYNLT3	3	1.97
NDRG1	3	1.96
MME	3	1.75
FGFR1	3	-1.40
ARNT2	3	-1.80
CXCL12	3	-2.16
MSX2	3	-2.22
MLLT11	3	-2.42
CDH2	3	-2.49
MMP11	3	-3.70
SFRP4	3	-3.84
MMP7	3	-4.13
