# Supplementary material for: A systematic review of transcriptomic studies of the human endometrium reveals inconsistently reported differentially expressed genes
Source: Reprod Fertil. 2023 Jul 7;4(3):e220115. doi: 10.1530/RAF-22-0115 (PMC10388686; doi:10.1530/RAF-22-0115)
Supplement: Table S3. Commonly reported differentially expressed genes common to ≥4 studies in studies comparing mid-secretory vs early secretory endometrium, and their average fold change in expression. [file supplementary_table_3.pdf]

Table S3

Gene Name	Number of studies reporting DEG	Average fold change (log_2)
IL15	7	2.25
PAEP	5	7.58
GPX3	5	5.64
DKK1	5	4.95
CLDN4	5	4.38
GADD45A	5	2.94
EDNRB	5	2.87
APOD	5	2.76
SERPING1	5	2.74
COMP	4	6.88
DPP4	4	5.99
SCGB2A2	4	5.91
C4BPA	4	5.60
TCN1	4	4.95
RBP4	4	4.80
CD55	4	4.75
MAOA	4	4.23
SOD2	4	3.64
MT1F	4	3.54
LAMB3	4	3.54
EFNA1	4	3.21
THBD	4	2.90
KRT7	4	2.70
GNLY	4	2.62
SLPI	4	2.31
ID4	4	2.24
MSX1	4	-1.92
EDN3	4	-2.60
