# Supplementary material for: A systematic review of transcriptomic studies of the human endometrium reveals inconsistently reported differentially expressed genes
Source: Reprod Fertil. 2023 Jul 7;4(3):e220115. doi: 10.1530/RAF-22-0115 (PMC10388686; doi:10.1530/RAF-22-0115)
Supplement: Table S4. Commonly reported differentially expressed genes common to ≥2 studies in studies comparing mid-secretory endometrium from ovarian stimulation-treated participants vs controls and their average fold change in expression, and their average fold change in expression. [file supplementary_table_4.pdf]

Table S4

Gene Name	Number of studies reporting DEG	Average fold change (log_2)
HAL	3	2.03
ID4	3	1.09
CD36	3	-1.72
GP2	2	10.25
MSX2	2	5.83
KCNJ2	2	4.82
EDN3	2	4.58
SORD	2	4.22
SLC15A2	2	2.82
DKK1	2	2.29
NUDT4	2	2.11
DF	2	2.09
DAF	2	2.01
SOD2	2	1.80
S100A4	2	1.72
CP	2	1.70
KRT23	2	1.56
CSPG2	2	1.50
MET	2	1.37
SERPINA3	2	1.32
TIMP3	2	1.22
ANXA4	2	1.21
IER3	2	1.19
ECGF1	2	1.17
GUCY1B3	2	1.14
TNFAIP2	2	1.04
PROS1	2	1.01
DDX52	2	1.01
RAB4A	2	-1.01
C11orf8	2	-1.05
CKB	2	-1.06
CYB5	2	-1.10
OFD1	2	-1.28
PIP5K1B	2	-1.53
KYNU	2	-1.81
TMOD1	2	-2.55
CDH13	2	-2.87
MYH11	2	-2.97
