# Supplementary material for: A systematic review of transcriptomic studies of the human endometrium reveals inconsistently reported differentially expressed genes
Source: Reprod Fertil. 2023 Jul 7;4(3):e220115. doi: 10.1530/RAF-22-0115 (PMC10388686; doi:10.1530/RAF-22-0115)
Supplement: Table S5. Commonly reported differentially expressed genes common to ≥2 studies in studies comparing mid-secretory endometrium from RIF patients vs controls, and their average fold change in expression. [file supplementary_table_5.pdf]

Table S5

Gene Name	Number of studies reporting DEG	Average fold change (log_2)
ANXA2	3	-0.99
NR4A1	2	4.27
AHSP	2	4.03
MADCAM1	2	4.03
SLC39A14	2	1.99
ANK3	2	1.89
FOSB	2	1.88
LATS1	2	1.52
TTNA57	2	1.44
BIRC3	2	1.42
FOLR3	2	1.30
FOLR1	2	1.28
PTK6	2	1.26
AIM1L	2	1.23
LRRC1	2	1.19
GATA2	2	1.15
ATF6B	2	1.10
AXIN2	2	1.09
BAIAP2	2	1.05
IKZF1	2	1.01
OR2M7	2	0.95
SCARNA9	2	0.95
CHN2	2	0.94
KALRN	2	0.65
SFTPD	2	-0.62
HMGA1	2	-0.67
TADA2A	2	-0.67
FGFR2	2	-0.74
NTNG2	2	-0.80
MTHFD1L	2	-0.85
LDHA	2	-0.88
PERP	2	-0.90
NCAM2	2	-1.07
SORBS1	2	-1.14
CCL23	2	-1.18
SOD2	2	-1.18
EDNRB	2	-1.32
FUS	2	-1.38
ASPN	2	-1.45
C4BPA	2	-1.57
IGHG1	2	-2.36
